# Pooled surveillance testing for asymptomatic SARS-CoV-2 infections at a Veterinary Teaching Hospital College, University of Minnesota, December 2020–April 2021

**DOI:** 10.3389/fpubh.2022.879107

**Published:** 2022-08-05

**Authors:** Janice Mladonicky, Addisalem Bedada, Colin Yoder, Kimberly VanderWaal, Jerry Torrison, Scott J. Wells

**Affiliations:** ^1^Department of Veterinary Population Medicine, College of Veterinary Medicine, University of Minnesota, St. Paul, MN, United States; ^2^Veterinary Diagnostic Laboratory, College of Veterinary Medicine, University of Minnesota, St. Paul, MN, United States

**Keywords:** COVID-19, SARS-CoV-2, surveillance, veterinary medicine, pooled testing, cost effective, serology

## Abstract

To evaluate the use of asymptomatic surveillance, we implemented a surveillance program for asymptomatic SARS-CoV-2 infection in a voluntary sample of individuals at the College of Veterinary Medicine at the University of Minnesota. Self-collected anterior nasal samples were tested using real time reverse transcription-polymerase chain reaction (RT-PCR), in a 5:1 pooled testing strategy, twice weekly for 18 weeks. Positive pools were deconvoluted into individual tests, revealing an observed prevalence of 0.07% (3/4,525). Pooled testing allowed for large scale testing with an estimated cost savings of 79.3% and modeling demonstrated this testing strategy prevented up to 2 workplace transmission events, averting up to 4 clinical cases. At the study endpoint, antibody testing revealed 80.7% of participants had detectable vaccine antibody levels while 9.6% of participants had detectable antibodies to natural infection.

## Introduction

The predominant strategy used to prevent viral transmission of SARS-CoV-2, the virus that causes COVID-19, in congregate settings has been implementation of public health mitigation measures, together with testing of symptomatic individuals with isolation following positive results. During the 2020 spring and fall semesters, the University of Minnesota (UMN) implemented compulsory COVID-19 mitigation measures which included mandatory facemasking, physical distancing, and reporting of symptoms followed by testing and mandatory isolation following positive test results. However, a symptomatic surveillance strategy may be inadequate to protect students and staff working and studying in clinical training programs wamongst s such as that at the College of Veterinary Medicine (CVM) at UMN. An alternative surveillance strategy for SARS-CoV-2 in congregate settings involves frequent testing of individuals without symptoms, a strategy referred to as asymptomatic surveillance. Recent estimates suggest up to 35% of all COVID-19 cases may be asymptomatic and the Centers for Disease Control and Prevention (CDC) estimates these individuals are 75% as effective at transmitting the disease as symptomatic cases (1, 2). Pooled testing by real time reverse transcriptase-polymerase chain reaction (rRT-PCR), in which multiple samples are mixed together following single RNA extraction may serve as a cost-effective strategy, optimizing diagnostic and labor resources, for asymptomatic active surveillance campaigns. Repeated use of pooled testing is commonly used as a cost-effective animal health surveillance strategy and has been considered in certain settings as a surveillance option by public health agencies. A report Yelin et,.al. showed that a single positive sample can be detected in pools of up to 32 samples using the standard COVID-19 RT-qPCR test, and another by Sunjaya et. al. showed a sensitivity of 96% in pools of 64 samples, which support efficient surveillance of populations (3–5). Another report demonstrated that the optimal pool size is 5 samples per pool when the individual prevalence is <10%, assuming retesting of individuals within test-positive pools to identify test-positive individuals and follow-up with health care providers (6). This report indicated a nearly 70% savings of reagents and personnel time.

The overall performance of the RT-PCR test using Bayesian Latent Class Methods in people during the first week of clinical signs has been estimated with test sensitivity of 68% and test specificity of 99% (7). The UMN Genomics Center developed a rapid-throughput SARS-CoV-2 PCR test was dwith analytic sensitivity demonstrated from 5 to 20 copies of the viral target per microliter (8). While the diagnostic sensitivity and specificity of this specific test has not been evaluated, performance of RT-PCR tests has been previously estimated using Bayesian Latent Class Methods in people during the first week of clinical signs, with estimated with test sensitivity of 68% and test specificity of 99% (7).

Simulation modeling may serve useful when performing a cost-benefit analysis of active surveillance in congregate settings. This can be particularly useful when the disease incidence in the community-at-large is low androutine testing may be expensive on a per case basis (9). In congregate settings where there is repeated and prolonged contact amongst individuals working or living in close proximity, modeling studies have demonstrated a potential benefit of routine PCR screening for asymptomatic individuals with isolation of PCR-positive individualsto reduce clinical cases (10). Although these findings may be influenced by whether a workplace is using PCR screening as a tool during the prevention, early mitigation, or widespread stages of an outbreak, evidence suggests asymptomatic surveillance can reduce workplace transmission and limit the number of clinical cases, supporting continuity of work or education (9, 11). Paltiel et. al. found screening every 2 days using a rapid, inexpensive, and even relatively insensitive sensitive (>70%) test, coupled with strict behavioral interventions to keep the effective reproduction number (Rt) <2.5, was estimated to maintain a controllable number of COVID-19 infections and permit the safe return of students to campus (12).

Unavoidable close contact amongst the CVM community and the occurrence of asymptomatic transmission makes frequent pooled testing, along with other public health mitigation measures, a potentially cost-effective strategy to mitigate transmission in high density settings. The objective of this study was to evaluate the feasibility and effectiveness of asymptomatic surveillance using pooled testing of students, faculty, and staff in reducing the incidence of COVID-19 in a veterinary medical college setting. To achieve this objective, participant surveys, cost analysis, nasal swab and serologic testing, and simulation modeling were employed to evaluate the program impact on acceptability, practicality, accuracy, and incidence, respectively.

## Materials and methods

### Study population

The University of Minnesota Veterinary Medical Center comprises several hospitals for small and large animals, employing 84 clinicians and 107 veterinary technicians at full time status during the study period. During the same time, the Veterinary Diagnostic Laboratory employed about 12 faculty and 91 technicians at full-time equivalent status. Veterinary student enrollment was 397 students. Veterinary students and instructors with in-student contacts at the CVM during the period from December 2020 to April 2021 were invited to voluntarily participate in this study with initial capment at 200. At the study's midpoint, enrollment was reopened to allow additional voluntary study participants. Participants eligible to participate were faculty members (including clinicians), veterinary technicians, laboratory technicians, graduate students, and others with on-going regular or intermittent contact with students at the Veterinary Medical Center. Study participants were recruited through a college-wide email to invite voluntary participation, following webinars given to provide information about the potential value of asymptomatic surveillance to prevent transmission to others. Study participants agreed upfront to submit nasal swab samples for biweekly testing, and to follow university guidelines after testing positive, including isolation from the university for 10 days. Symptomatic participants were instructed to submit their sample upon return to campus. At the study outset, participants provided information related to their current age, University of Minnesota status (student, faculty, staff, and graduate student), and travel out of state in the past month. Individuals information was kept confidential and used only for data analyses. The 7-day rolling average of COVID-19 cases in our study population, proceeding and following the study period, reported cases per capita for December 1, 2020, and May 6, 2021, were 79.2 and 24.8 cases per 100,000 per day respectively^1^. In order to estimate the infection rate within our sample population at the CVM, we queried participants on their disease status proceeding the study onset, during the study with sample submissions, during the final survey, and performed antibody testing near the study's conclusion.

### Sample collection for SARS-CoV-2 PCR testing

The study team developed and shared written instructions and an instructional video demonstrating the self sampling and submission procedure. Participants submitted nasal swabs (Sterile Polyester Spun Swab, SteriPack USA, LTD, 60564RevA) from the anterior nares for testing on Mondays and Thursdays from December 10, 2020 until April 8, 2021. Nasal samples were self-collected by study participants using a defined protocol (16). Multiple sampling kits were provided to the participants at multiple points during the study: home sampling was encouraged. If a participant absent from campus on submission day, they were instructed to submit upon their return for the sample to be collected during the next scheduled submission. Samples were placed in tubes containing 3 ml of DNA/RNA Shield from Zymo Research Corp (17). Sample submission forms were submitted with each sample. Sample tubes, individually identified using bar-coded labels, were submitted to collection locations at the CVM, processed for submission, and then transported and submitted to the UMN Genomics Center for day of collection testing.

### SARS-CoV-2 RT-PCR testing

Testing was conducted using a modified protocol by Nelson et,.al. Nasal swab samples were randomly pooled in groups of a maximum of 5 samples per pool (8). The final pool each week consisted of all remaining samples, creating a final pool of 1–4 samples at each sample date. To compare the performance of the pooled results, Individual samples were also tested at 3 separate sample submission events for comparaison. Anterior nares swab samples in 3 mL DNA/RNA Shield were first heat-inactivated at 56°C for 20 min. 25 uL from each of 5 samples were transferred to a 96-well-deep well-plate containing 94 uL of Lysis Buffer (4.67 M GuHCl, 5.8 mM TCEP, 23.3 mM EDTA, 23.3 mM Tris, pH 7.0, 0.23 % (v/v) Igepal CA-630, 100 units/mL Proteinase K) and pipette-mixed 10 times. If testing individual samples, 100 uL of sample and 75 uL of lysis buffer were used. The plate was sealed and again incubated at 56°C for 20 min.

Following lysis, 175 μL of a SPRI bead solution (1:50 dilution of 3X washed Cytiva Sera-Mag SpeedBeads™ 65152105050250, 18% (w/v) PEG-8000, 1M NaCl, 10 mM Tris-HCl, 1 mM EDTA) was added to the lysate and pipette-mixed 10 times, incubated at room temperature for 10 min, and pipette-mixed an additional 10 times. Following incubation and mixing, the plate was placed on a magnet and the magnetic beads were allowed to pellet, after which the supernatant was removed, and the beads were washed twice with 800 μL freshly prepared 80% ethanol. Beads were allowed to dry off-magnet for 10 min at room temperature, and then were resuspended with 50 μL nuclease-free H20. After magnetizing again, the clear supernatant was removed to a fresh plate, and this solution was used as input for RT- qPCR. Real-time reverse transcription PCR was then performed, as described by Nelson et,.al using CDC assay sequences for nucleocapsid 1 and 2 (N1/N2) and RNase P (RP) in a triplex reaction with a FAM probe for N1/N2 and an ATTO probe for RP which can be found at this web address: https://www.cdc.gov/coronavirus/2019-ncov/downloads/rt-pcr-panel-primer-probes.pdf (8). Each PCR run contained both a positive and negative control.

### Results interpretation

Pooled and individual samples were classified positive, negative, or invalid considering the following cycle threshold (Ct) values:

N1/N2 Ct = 37 and RP Ct < 40 Positive.

N1/N2 Ct < = 37 and RP Ct < 36 Negative.

RP Ct either undetected or > 40 Invalid (quantity not sufficient).

If the nasal swab pool tested negative, then all individuals (samples) in the pool were considered as non-infected. If the pool tested positive, then the laboratory retested the individual samples within the positive pool to identify the test-positive individuals.

### Result reporting

Test results were provided to individual study participants *via* a secure email center, powered by Proofpoint^®^ software, allowing for the encryption of Protected Health Information. Individuals that tested positive were immediately informed to contact a health care provider for further medical care, and follow UMN guidance for COVID-positivity, including 10 days of self-isolation. Study team investigators reported positive testing individuals to the Minnesota Department of Health for community contact tracing.

### Sample collection for SARS-CoV-2 antibody detection

All enrolled study participants were invited to submit a sample for IgG antibody detection. Samples were self-collected, one time, using Neoteryx Mitra^®^ 10 μl samplers by volumetric absorption of capillary blood from a finger-stick at the study's conclusion. Samples accompanied with a brief vaccine status questionnaire were submitted to study investigators and then mailed to Quansys Biosciences Laboratory for testing.

### SARS-CoV-2 antibody detection

Samples were tested using the Quansys Q-Plex™ SARS-CoV-2 Human IgG (5-Plex) with established protocols (18), a semi-quantitative chemiluminescent ELISA that concurrently measures Human IgGs reactive to SARS-CoV-2 spike subunit S1, spike subunit S2 and nucleocapsid (Nc). The positive cut off indexes were >7.7 U/ml, >30 U/ml, and >25 U/ml for spike S1, spike S2 and Nc, respectively. Samples positive for spike subunit S1, spike subunit S2 and Nc were considered natural viral infections and may also have had vaccine exposure. Samples positive for spike S1 and negative for Nc were considered as vaccine exposure only. Individuals negative to all three proteins were considered without natural or vaccine exposures.

### Sample submission forms and surveys

#### Sample submission forms

Participants completed and submitted a questionnaire detailing identifying information, dates of sample collection and submission, exposure and travel history, and symptoms with all PCR and antibody sample submissions. Vaccine information was collected for all antibody test submissions. Submission rates by participant type and primary work location, and participant benefits of the system were evaluated.

#### Initial, case positive, and final surveys

An electronic survey was sent to all consenting participants *via* electronic mail using Qualtrics Survey Software (Provo, UT) upon study onset and completion. A separate voluntary survey was sent to those participants who tested positive through PCR testing from this study. All surveys utilized a semi-structured format to capture demographic and temporal risk factors for COVID-19. The final survey captured additional information including COVID-19 disease and vaccination status and information to evaluate system acceptance and feasibility with 4-point Likert type scale and multiple-choice questions.

### Statistical methods

Data were manually reviewed and validated, and major qualitative findings were described and summarized using frequency distributions based on thematic areas to supplement quantitative findings (Microsoft Excel 2021).

### Modeling

A previously described stochastic compartmental model by VanderWaal et,.al. assuming homogeneous mixing, was used to simulate the spread of SARS-CoV-2 in the College (9). Briefly, this model accounted for transmission amongst pre-clinical, clinical, and asymptomatic students/staff/faculty (determined by the effective reproduction number at the workplace) and incorporated a constant background rate of community-acquired infections. Parameters that determine the rate at which individuals transition between compartments are defined in Table 1.

**Table 1:** Model parameter estimates.

**Parameter**	**Plausible range**	**Sources**
Reproduction number	1.0–3.0	[27,28]
Relative infectious undetected carriers	0.75	[29]
Background transmission rate (new cases per 100 k per day	10–500	[27]
Percent asymptomatic (*U* class)	0.3	[29]
Latent period	5	[36, 37]
Pre-symptomatic period	1.5	[38, 39]
Clinical (infectious) period	6	[38]
Prop. people that go to work when ill	0.3	[27, 40]
Home quarantine period (default = 10 days)	10	CDC
Sensitivity of daily temperature screening	0.7	[41, 42]
Frequency of PCR testing (interval in days)	3	
Delay in results for PCR test	1	
Sensitivity of PCR testing	90%	[41]

The baseline model employs daily symptom-based screening, wherein clinically infected students/staff are detected with some probability (based on estimates of the sensitivity of temperature-based screening, Table 1); no routine PCR-testing is performed; thus all exposed, asymptomatic and pre-clinical individuals will continue to attend work/school. In the bi-weekly testing scenario evaluated as a comparison, testing is employed every 3 days, and any individual in the asymptomatic, pre-clinical, or clinical class is detected with an 90% probability (based on the approximate sensitivity of PCR tests). A one-day delay in PCR results is assumed. Any individual that is identified as a case remains at-home for a 10-day period. In both the baseline and bi-weekly testing scenarios, there is no contact tracing of positive cases, therefore results represent worst-case scenarios. Given that this study evaluated the potential use of bi-weekly testing to avert a workplace outbreak, we assumed that there were no infected students/staff/faculty at the beginning of the simulations, thus virus could only be introduced by community-acquired infections. We also assumed a population size of 100 workers, with all participants classified as susceptible at the onset of the simulation; previous results showed that results were consistent across population sizes (9).

**Figure 1:**
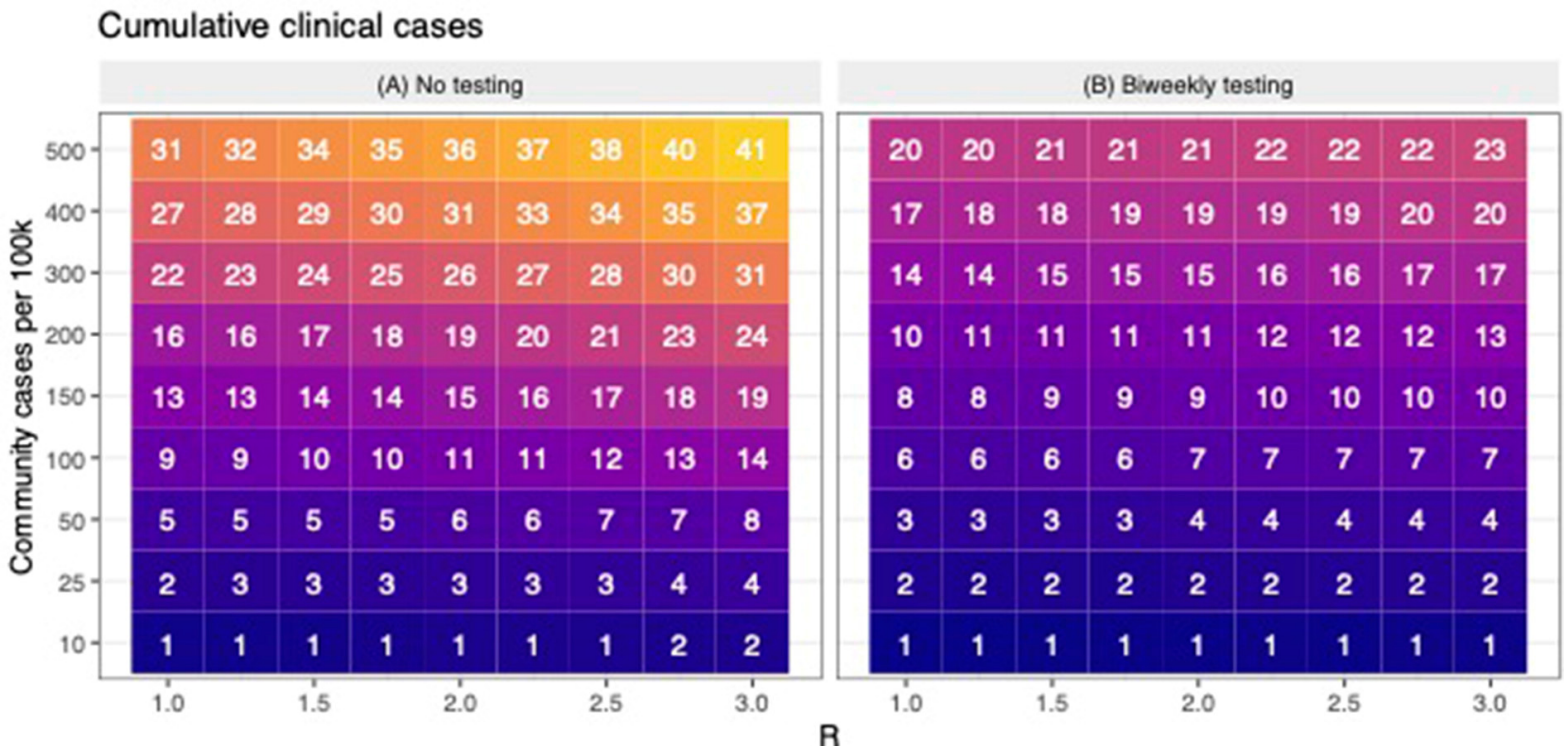
The cumulative number of expected clinical cases when there is no testing and during bi-weekly testing. **(A)** shows the predicted number of cumulative clinical cases (in a population of 100) across scenarios with variable levels of workplace R and community transmission. For the same levels of workplace R and community transmission as **(A,B)** shows the predicted number of cumulative clinical cases when bi-weekly testing was employed. Across **(A,B)**, lower to higher numbers are shaded in darker to lighter colors, respectively.

**Figure 2:**
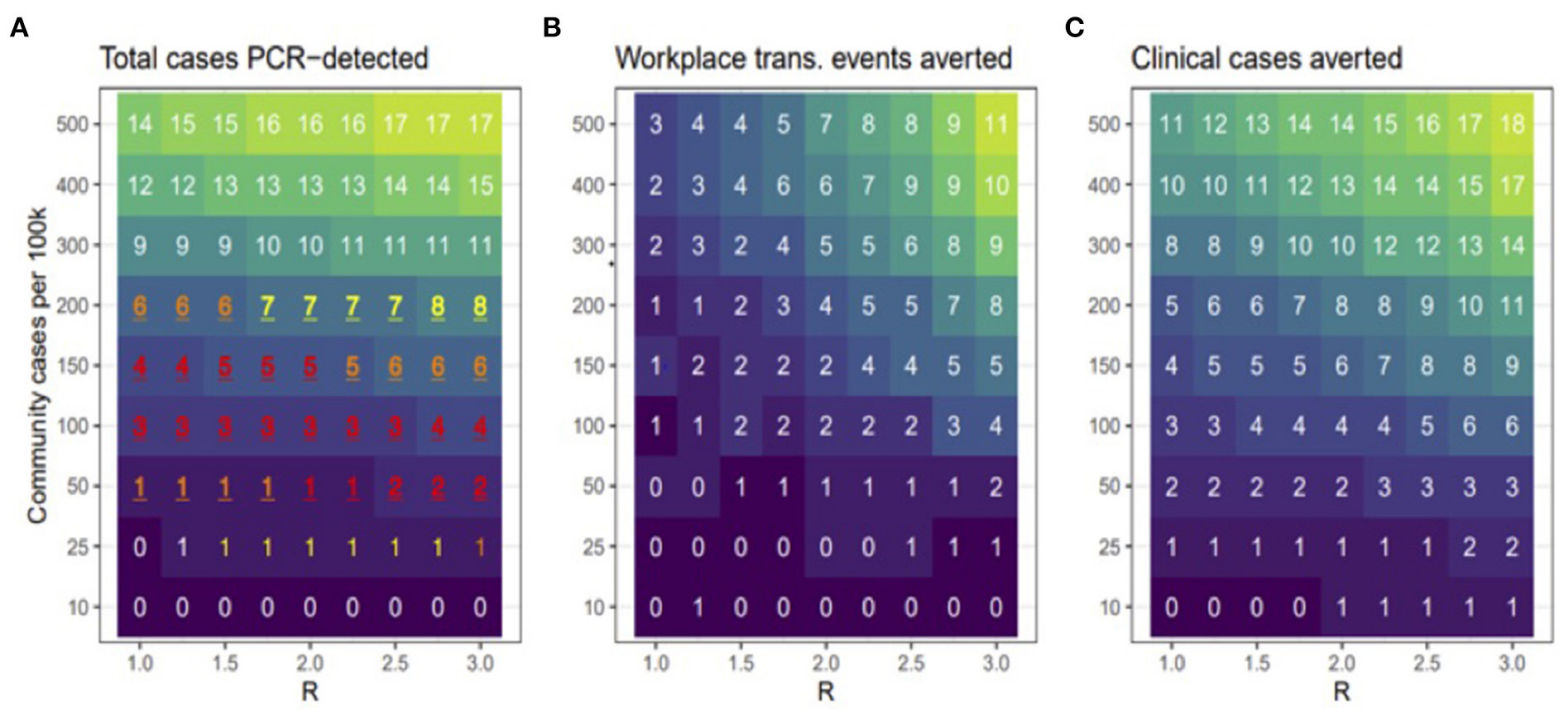
Model scenarios. **(A)** shows the total number of cases that were PCR-detected (clinical or non-clinical). Red, orange, and yellow text shows scenarios in which the 95% prediction interval overlapped the observed number (3) of PCR-positive cases in the cohort, with redder text indicating better correspondence between modeled and observed results. **(B)** shows how many workplace transmission events were avoided relative to the no testing baseline and **(C)** shows the total number of clinical cases averted during bi-weekly testing relative to no testing baseline. Across all panels, lower to higher numbers are shaded in darker to lighter colors, respectively.

Based on previous work, the cumulative number of cases estimated by the model is highly sensitive to assumptions about the rate at which participants become infected outside of work/school (i.e., community-acquired infections) and the workplace transmission rate (workplace R), which determines how quickly the virus spreads amongst participants once introduced (9). Thus, we evaluated the number of clinical cases averted (i.e., cumulative number of clinically infected individuals in the baseline scenario minus cumulative number of clinically infected individuals in the bi-weekly scenario) across a range of values for workplace *R* (1–3) and rates of community-acquired infections (10–500 new cases per 100 k per day). Model results were not found to be particularly sensitive to other model parameters, therefore these were held constant (Figures 1, 2) (9).

The range of workplace *R* was based on literature. The range for rate of community-acquired infection rates is based on CDC indicators of low and high community transmission, as well as the number of new cases observed per 100 k per day in Ramsey County during the study period (which peaked at >90 per 100 k). We set our high-level to 5x the threshold for high community transmission, assuming substantial underreporting of mild or asymptomatic cases. We ran 1,000 simulations for each scenario (baseline and bi-weekly testing for each combination of workplace R and community transmission rates), with the cumulative number of clinically infected individuals summarized over 90 days. The number of averted cases was calculated as the difference between the median number of clinical cases in bi-weekly testing scenarios relative to the baseline scenarios. The model was coded in R statistical software v3.6.0 (19).

## Results

### Study population

**Figure 3:**
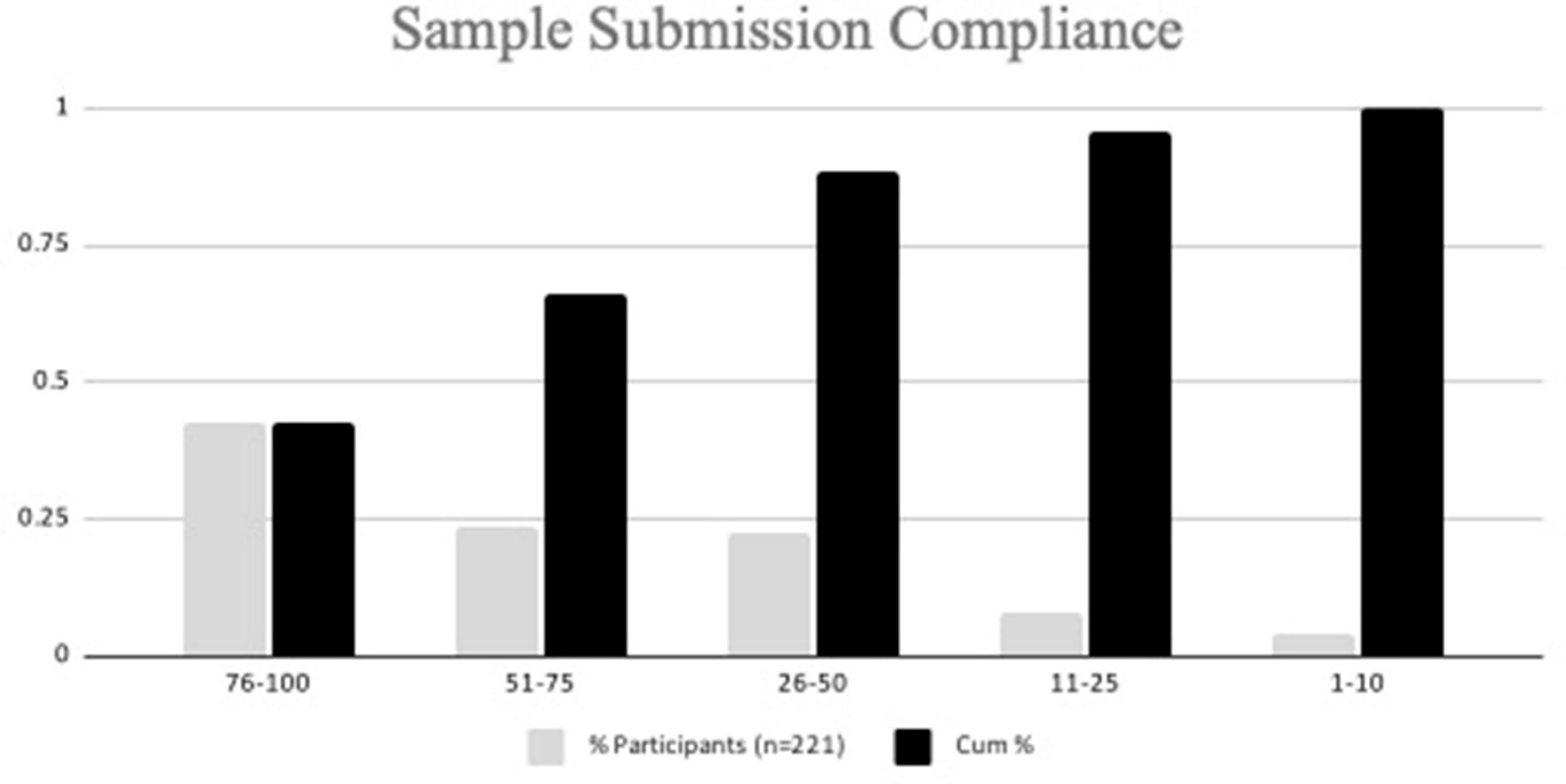
Sample submissions among participants.

Of the 230 participants that signed up for the study, a total of 221 participants submitted at least one nasal swab sample for testing during the study (Figure 3). Nine participants signed up for the study but did not submit a sample and were excluded from the total. The majority of participants were faculty (24%) and veterinary technicians (28%); 22% were veterinary students (Table 2).

**Table 2:** Sample submissions.

**Occupation**	**Count of Participants (%): nasal swab PCR sampling**	**Count of Participants (%): antibody test sampling**
Years 1–3 veterinary students	37 (16.7%)	22
Year 4 veterinary students	11(4.9%)	5
Interns, residents, and graduate students	31 (14.0%)	18
Faculty	54 (24.4%)	41
Veterinary technicians	61 (27.6%)	41
Other (staff, laboratory personnel, researchers)	27 (12.2%)	18
Total	221	145

#### Survey results

The response rate for 4 different surveys distributed over the course of the study ranged from 71 to 100% (Table 3). Participants were repeatedly queried on COVID-19 symptoms, test positivity, and vaccination status as shown in Table 3. Of those participants who completed the first survey, 63 (33.7%) had experienced clinical signs consistent with COVID-19 since January 1, 2020, and 5 (2.7%) had previously tested positive for COVID-19 before enrolling into the study. Of the 4501 accompanying submission forms collected with samples during the study period, 0.2% (10/4501) self-reported having COVID-19 compatible symptoms within the previous 7 days. Of the 221 participants who submitted a nasal sample for PCR testing, 71.0% (157/221) completed the final survey, which indicated that 15.4% (24/156) of study respondents reported experiencing COVID-19-related symptoms from December 2020 to April 2021. Most symptoms were reported to have occurred in January 2021 (9/24, 37.5%), followed by March (4/24, 16.7%) and April (2/24, 8.3%) of the same year. Two respondents (2/156) reported testing positive from an external testing source elsewhere on January 15 and 19, 2021.

**Table 3:** Attributes and responses of surveys distributed during the study period.

	**Surveys accompanying PCR sample submissions (*N* = 221, 4,525 samples)**	**Initial survey (*N* = 221)**	**Survey accompanying antibody sample (*N* = 145)**	**Final survey (*N* = 156)**
Time period for survey responses	December 2020–April 2021	January - December 2020	December 2020 - April 2021	December 2020 - April 2021
Response rate (*n*)	99.5% (4,501 surveys)	84.6% (187)	100% (145)	71.0% (157)
COVID 19 symptoms	4.5% (10 participants)	33.7% (63)	-	15.4% (24)
COVID-19 test positive	-	2.7% (5)	3.5% (5)	1.3% (2)
COVID-19 vaccination	-	^N/A*^	93.8% (136)^**^	93.6% (146)^***^

N/A* Vaccine not yet approved.

- Question not asked.

** Received at least one dose of vaccine series or single dose for 1 dose series.

*** Pfizer vaccine was most frequent (47.0%, 69/147) followed by Moderna (44.0%, 64/147), and Johnson & Johnson/Janssen (9.5%, 14/147).

### Antibody testing

A total of 145 participants collected and submitted a blood sample for antibody testing between 4/8/2021 and 4/19/2021. From the associated antibody test submission survey, 136 (93.8%) of the participants indicated they had already received at least one dose of the COVID-19 vaccine, 91 (62.8%) had already received all scheduled doses of vaccine, and 42 (29.0%) had received only the first dose of vaccine prior to blood collection. Five (5/145) self-reported they had previously tested positive for COVID-19 at the time of antibody testing(one was identified in our study while the others tested elsewhere) (Table 3).

Of the 145 serum samples analyzed using the Quansys Q-Plex™ SARS-CoV-2 Human IgG ELISA, 132 (91.0%), 120 (82.7%) and 17 (11.7%) serum samples were positive for S1, S2 and Nc, respectively. The results of 117 (80.7%) study participants revealed detection of vaccine antibody (positive for S1 and negative for Nc) and 14 (9.6%) detected antibody (positive for Nc, S1 and S2) to SARS-CoV-2 field virus from natural infection. The serum analysis of 12 (8.3%) participants showed antibody level less than the cut-off for S1, S2, and Nc, indicating test-negativity for antibodies to both vaccination and natural exposure. Two (1.4%) samples were uniquely positive for S1 and Nc but negative for S2 and considered inconclusive. One sample (0.7%) was positive for Nc and negative for S1 and S2. Of the 14 samples considered positive for natural infection, only three (21%) indicated having a previous SARS-CoV-2 positive test. Further, 2 of the five participants that indicated a previous positive test result were not antibody test-positive for natural infection. Although 136 of the participants received at least one dose of the COVID-19 vaccine, only 117 (86.0%) had detectable antibodies indicating vaccine exposure.

### PCR sample submissions and result reporting

In total, 4,525 total samples were submitted, with a mean of 20.3 samples per participant. Out of a total of 33 sample submission opportunities, 66.1% of participants submitted at least 51% of the maximum number of samples (Figure 3). Because the sample media, DNA/RNA Shield ™, maintains sample integrity for a minimum of 30 days when stored at ambient temperatures (4–25 degrees C), participants were permitted to collect and submit their sample on different days. Days of sample collection to sample submission ranged from 0 to 51 days. Seventy-one percent (3,221/4,525) of samples were submitted on day of collection, 773 (17.1%) samples were submitted 1 day post collection, 471 (10.4%) samples were submitted 2–10 days collection, and 60 (1.3%) samples were submitted >10 days post collection. The time from sample collection pickup, laboratory submission, and return of testing results, including those from disassembled pools (if indicated) to study investigators was 10–139 h (median 34 h). Once results were released to study investigators, the participants were notified of their results typically within 1 h.

### PCR test results

#### Pooled testing

Overall, 921 sample pools were tested, spanning 18 weeks, from 4,525 nasal samples collected at 33 sample submission events. Pooled samples were deconvoluted for individual testing for comparative pools (*n* = 229 from 3 sampling events), invalid pooled results (*n* = 5), and for positive pools (*n* = 3) (Table 4). Ultimately, three pools were test positive (0.3%), 3 were classified invalid, and 915 were classified negative (99.3%). Overall, 104 pools were deconvoluted and 513 swabs were analyzed individually. All individual samples within comparative pools remained negative. Upon deconvolution of the invalid pools, only one individual sample remained invalid, with the remaining samples classified as negative (Table 4). Testing the individual samples within 3 test-positive pools identified one sample positive from each pool, resulting in an overall observed prevalence of 3/4,525 (0.07%). Positive samples were identified on 01/11/2021, 01/14/2021, and 01/28/2021 (Table 4). Two of the three test-positive participants did not self-report COVID-19 compatible symptoms prior to sample collection and submission. In our study, this pooling method prevented 4,012 (4525-513) tests from being performed. All test-positive samples were collected within 1 day of submission.

**Table 4:** Description of deconvoluted pools with Ct values for positive pools and individual samples.

**Deconvoluted pools, 2020-2021**									
**Sampling date**			**Pooled samples**			**Individual tested samples**			
	**Total samples (pools)**	**Deconvoluted pool count (individual samples)**	**Reason**	**N1/N2 (Ct)**	**RP (Ct)**	**Sample#**	**N1/N2 (Ct)**	**RP (Ct)**	**Result**
17-Dec	151 (31)	2 (10)	Invalid pool	ND	ND	Samples 1–4	ND	≤ 36	Negative
						Sample 5	ND	ND	Invalid
			Invalid Pool	ND	ND	Samples 1–4	ND	≤ 36	Negative
						Sample 5	ND	ND	Invalid
21-Dec	160 (32)	32 (160)	COMP	ND	ND	Samples 1–159	ND	≤ 36	Negative
						Sample 160 (pool 24)	ND	ND	Invalid
7-Jan	160 (32)	0^*^	Invalid pool	ND	ND	Not performed (15 total samples affected)			
11-Jan	147 (30)	1 (5)	Positive pool	31.8	25.3	Samples 1-4	ND	≤ 36	Negative
						Sample 5	29.9	25.5	Positive
14-Jan	145 (29)	1 (5)	Positive pool	21	24.9	Samples 1–4	ND	≤ 36	Negative
						Sample 5	20.6	24.6	Positive
22-Jan	152 (31)	31(152)	COMP	ND	ND	Samples 1–152	ND	≤ 36	Negative
28-Jan	167 (34)	1 (5)	Positive pool	23.7	24.6	Samples 1–4	ND	≤ 36	Negative
						Sample 5	21.3	24.8	Positive
11-Feb	166 (34)	34 (166)	COMP	ND	ND	Samples 1–166	ND	≤ 36	Negative
15-Mar	138 (28)	1 (5)	Invalid pool	ND	ND	Samples 1–5	ND	≤ 36	Negative
22-Mar	120 (24)	1 (5)	Invalid pool	Invalid pool	ND	Samples 1–5	ND	≤ 36	Negative

* On primary execution, 4/32 pools were inconclusive (RP not detected). As pooled groups, these 4 pools were retested, and 3/4 pools remained inconclusive. Samples were discarded and individual sampling was not performed.

COMP, Comparison Pool; ND, Not Detected.

#### Test-positive survey

All test-positive participants were invited to complete a follow-up survey, and two of the three eligible study participants completed the survey. Both respondents indicated having received COVID-19 test-positive results from an external testing source on the day following study sample submission. One became symptomatic for COVID-19 2 days before sample submission (though not indicating this on study sample submission) while the other positive respondent became symptomatic the day after they submitted their sample.

#### Final survey

Participants were queried on their motivations for participation and overall acceptance of the study. A plurality indicated they participated, “*out of concern for unknowingly transmitting COVID-19 to their family and friends”* and wanting “*to do their part by participating in efforts to help keep the community as safe as possible during the pandemic* [81.0% (126/156) and 80.1% (125/156)] respectively.” Participants were asked about whether knowing test results twice weekly alleviated any distress caused by the pandemic. Many responded they were “*better able to focus attention on clinical/academic/research work* (35.0%, 55/157)*,” and* felt “*more comfortable being on campus for work or training* (58.3%, 91/156).” Further, most reported having “improved confidence of not transmitting the virus to others in the workplace [75.2% (118/157) or in home or social settings (73.7%, 115/156)] when knowing test status twice weekly. Sixty-nine percent (69.2%, 108/156) of survey respondents indicated they definitely would participate in a similar future asymptomatic surveillance study.

### Cost of surveillance

During the study period, 936 PCR tests were performed (not including the comparative individual sample tests), including 921 pooled samples and 15 individual samples from the 3 test-positive pools after splitting into individual samples. This approach prevented the use of 3,589 tests. The cost of pooled testing including repeat testing of individual specimens from positive and suspect pools was $14,040 compared to $67,875 for individual tests resulting in a cost savings of $53,835 (79.3%). Supply costs (reagents, consumables), testing costs, and labor costs (kit preparation and sample processing) required for pooling tests were $33,248, $14,040 and $4,612 respectively. The cost of collecting and testing (supplies, labor, and tests) was about $11.47 per sample ($51,899 / 4,525 individual samples).

### Modeling

In this study, we used simulation modeling to assess the effectiveness of biweekly PCR-testing in reducing COVID-19 circulation within CVM across a range of scenarios. Across a range of assumed values of workplace *R* and community transmission, the model suggests that testing bi-weekly reduced the number of clinical cases up to 50% (Figure 1). Our observed data revealed that all the 3 positive cases occurred in January, when case rates in Ramsey County, MN were peaking. Figure 2A shows that the scenarios yielding ~3 positive PCR tests (95% prediction windows included 3) generally had community transmission rates of 100 cases per 100 k, which is well-aligned to the CDC case counts for Ramsey County reported during this period. By focusing on scenarios that produced predictions that were consistent with the observed number of PCR-positives (shown in red text in Figure 2A), our model results suggest biweekly testing prevented about 1–2 workplace transmission events in our study population (Figure 2B) and averted 3–4 clinical cases per 100 people (Figure 2C). While these numbers are low, the absolute number of cases averted through bi-weekly testing is more striking for the scenarios that produced larger outbreaks (with high workplace R and community transmission, Figure 3).

## Discussion

Universities across the United States have approached COVID-19 testing strategies in multiple ways, from simple encouragement for testing if symptomatic to mandatory testing for all students (20). Despite the myriad of testing strategies implemented across institutions, positive COVID-19 test results for college aged adults spiked at the start of most fall 2020 semester classes indicating that University settings need robust, cost effective COVID mitigation strategies (6). Previous studies have modeled the effects of symptomatic and asymptomatic surveillance strategies (12, 21–23), alternative testing strategies to increase throughput (24), and investigated the outcomes of outbreaks and the response on campuses (25, 26). Comprehensive testing strategies for both symptomatic and asymptomatic cases have been reported by Hamer et al. (27), with pooled samples being used in multiple instances (28, 29). This study expands on previous work by implementing a more cost-effective surveillance strategy, including antibody testing to characterize the study population, and modeling potential community spread to validate the screening program effectiveness.

Overall, our study population was low risk for disease transmission, likely the result of COVID-19 mitigation protocols already in place together with a highly altruistically motivated study population who self-selected to participate. The study conclusion of high self-reported vaccination rate against COVID-19 was validated by antibody testing. Unexpected, was the low participation rate among veterinary students. We speculate anxiety related to testing positive and the need for isolation may have contributed to the unexpectedly low participation rate among veterinary students, especially those in their 4th (clinical) year of training. Employees (both regular and student) were eligible for paid leave for quarantine or isolation due to COVID-19. Veterinary students, however, who rotate through clinical rotations during the 4th year of their training, were not eligible for time off if they missed a rotation and were required to complete rotations virtually or make them up later. The potential for a missed rotation due to isolation leading to a delayed graduation may have influenced low compliance among this group.

It has been well-recognized that infected persons who remain asymptomatic play a significant role in the transmission of SARS-CoV-2. Hence, detection and isolation of asymptomatic COVID-19 patients and tracing of close contacts is one important strategy to lessen the magnitude of the problem. By comparing our results with the expected number of cases, based on modeling, we demonstrated that our asymptomatic testing strategy prevented workplace transmission events and averted clinical cases within the CVM. The model also suggested that biweekly testing would be most beneficial when community transmission is high and with increasing testing frequency, additional cases could be averted. This model can be applied to other similarly sized congregate settings such as other universities.

A limitation of this surveillance study was its voluntary nature, which likely contributed to a underrepresentation of infections in our sample population. While antibody testing at the study conclusion was conducted to help determine the infection status of our study population, it too was voluntary to participants. The true infection rate within our sample population remains unknown because of confidentiality agreements within the college. However, infection rates are likely to have been low as a result of exclusion of COVID-19 exposed or symptomatic individuals from the college until quarantine/isolation periods lapsed, as required by university-wide public health guidelines.

Only three of the 14 study participants positive for SARS-CoV-2 natural infection based on antibody testing had self-reported a previous positive test result. Several reasons may explain this. While it is possible these participants may represent undetected infections from our PCR study, other explanations are possible, including that these participants did not submit a sample during the time of infection, did not disclose a positive test conducted elsewhere, had a previous unknown case of COVID-19, or were not naturally infected with SARS-CoV-2 but another coronavirus that cross-reacted with the nucleocapsid protein. Interestingly, two of the five respondents from the antibody survey who self-reported a previous infection (diagnosed 7/30/2020 and 1/19/2021), were considered negative for natural infection based on antibody testing. While the duration of immunity following COVID-19 vaccination is ongoing, previous studies have suggested high vaccine effectiveness would remain at least 5 months following vaccination, a time frame longer than our study period (30, 31).

In a low risk setting with other public health mitigation measures in place, this study demonstrates the feasibility, acceptability, and effectiveness of implementing a cost-effective asymptomatic surveillance pilot program, utilizing repeated frequent pooled testing, in a congregate setting where physical distancing is unavoidable.

## Data availability statement

The anonymized raw data supporting the conclusions of this article will be made available by the authors, without undue reservation.

## Ethics statement

The studies involving human participants were reviewed and approved by University of Minnesota. The patients/participants provided their written informed consent to participate in this study.

## Author contributions

SW devised the project providing the main conceptual ideas. JM and AB were lead authors of the manuscript. JM, AB, and SW worked out the logistical details and collected data. SW, JM, AB, CY, KV, and JT discussed and analyzed the results and contributed to the final manuscript. All authors contributed to the article and approved the submitted version.

## Funding

Funding was provided by the University of Minnesota Medical School, Office of the Vice Dean for Research.

## Conflict of interest

The authors declare that the research was conducted in the absence of any commercial or financial relationships that could be construed as a potential conflict of interest.

## Publisher's note

All claims expressed in this article are solely those of the authors and do not necessarily represent those of their affiliated organizations, or those of the publisher, the editors and the reviewers. Any product that may be evaluated in this article, or claim that may be made by its manufacturer, is not guaranteed or endorsed by the publisher.

## Footnotes

^1^Population of Ramsey County (location of veterinary college): 550, 321 (US Census Bureau) (13), 7 day rolling average of COVID-19 cases from 11/25/2020 - 12/1/2020 was 435.7 and the 7-day rolling average of COVID-19 cases in Ramsey County from 4/30/2021-5/6/2021 was 16.426 (Source: Minnesota Department of Health based on population of Ramsey County, CDC COVID Data Tracker) (14, 15).

## Abbreviations

CDC: Centers for Disease Control and Prevention
CVM: College of Veterinary Medicine
COVID-19: Coronavirus disease 2019
DNA: Deoxyribonucleic acid
ELISA: Enzyme-linked immunosorbent assay
Ig: Immunoglobulin
R: Basic reproductive number
RNA: Ribonucleic acid
RT-PCR: Real- time reverse transcription-polymerase chain reaction
SARS: Severe acute respiratory syndrome
UMN: University of Minnesota.
